# An elastic fiber based on phononic crystals

**DOI:** 10.1038/s41598-021-98854-4

**Published:** 2021-09-28

**Authors:** Farzaneh Motaei, Ali Bahrami

**Affiliations:** Optoelectronics and Nanophotonics Research Laboratory (ONRL), Faculty of Electrical Engineering, Sahand University of Technology, Tabriz, Iran

**Keywords:** Condensed-matter physics, Acoustics

## Abstract

In this study, a novel elastic phononic crystal fiber has been presented for the first time. This proposed structure can expand the sonic communications field, significantly. In order to realize the elastic fiber performance, solid–solid phononic crystal has been utilized. The phononic crystal structure operates as cladding in surroundings and central region acts as core of fiber by elimination of rods. Incident acoustic waves with transverse polarization have confined and propagated in the core region of the phononic crystal fiber. Two types of phononic crystal fiber with different core radii have been investigated. Incident elastic waves can confine in the core region with confinement factor higher than 500. Also, longitudinal losses have been achieved low and equal to 0.35 dB/km.

## Introduction

One of the important technological advances in the communication systems is the discovery of optical fibers in the early twentieth century^1^. The information in the optical fiber can transmit with a very high speed and low losses. The confinement and transmission mechanisms in traditional optical fibers are based on total internal reflection phenomenon occurred by placing the high refractive index material in the central region of fiber and low refractive index material in cladding. Some limitations of conventional optical fibers make the photonic crystal fibers become more attractive. Photonic crystal fiber (PCF) is a different type of fibers which was presented for the first time by Knight et al. in 1996^2^. Generally, photonic crystal fiber is formed of a single material such as silica which contains an air hole array running along fiber length. There are many different lattice patterns for air hole arrangement. Photonic crystal fibers are divided into two main classifications as index-guiding and photonic band gap-guiding fibers. One of the clear diversities between traditional and photonic crystal fibers is using a single material in photonic crystal fibers, instead of two different materials^1^. Photonic crystal fibers encouraged us to try to phononic crystal fiber designing.

Phononic crystals are the man-made structures which are dual of photonic crystals in acoustic field. These sonic structures are made similar to photonic crystals. Arrangement of a periodic array of scatterers in the background with different acoustic/elastic properties can produce the phononic crystals. Regarding to the scatterers and background materials, phononic crystals can be divided to three categories such as solid–solid, fluid–fluid, and solid–fluid structures. Periodic arrangement and high difference in acoustic/elastic properties between scatterers and background can make one or more band gaps in the device dispersion curve^3^. Acoustic waves with the band gap frequency have been blocked by periodic phononic crystal. This property makes phononic crystals become interesting for many engineering applications such as processing^4^, sensing^5^, etc. Many investigations in phononic crystals such as guiding^6,7^, filtering^8,9^, switching^10^, demultiplexing^11^, and sensing^12^ have been done in past two decades. In 2005, an investigation about elastic mode propagation along the photonic crystal fiber axis has been done^13^. Also, a fiber Bragg grating displacement sensing system to investigate the phononic crystal dynamic behaviors has been presented in 2018^14^. However, to the best of our knowledge, phononic crystal fibers have not proposed and designed yet. Although, there are some differences in the propagation nature, and polarizations of optical and acoustic/elastic waves, but designing of phononic crystal fibers is realizable. This novel device can present a new window in acoustic/elastic communication field. Since acoustic waves cover a wide spectral frequency range, so, their applications are wide, too. Thus, phononic crystal fibers as a new platform could be very useful in acoustic fields.

In this paper, we have tried to present a novel phononic crystal fiber structure for the first time. This phononic structure is formed by placing periodic tungsten rods in the Poly methyl methacrylate (PMMA) background with hexagonal lattice arrangement. The core region for phononic wave propagation has been built up by eliminating one or more tungsten rods in the central region of the fiber. At the first step, only central tungsten rod has been omitted, next, a single ring of rods in the vicinity of the central rod has been eliminated, too. Despite of the photonic crystals which handles transverse polarizations, only, phononic crystals can support both longitudinal and transvers modes. In phononic crystal fibers, the periodicity of scatterers is as radial, so transverse modes can only perceive the periodicity of core surrounding regions. On the other words, longitudinal waves can propagate parallel to the fiber length where there is not any periodicity. In addition to these, fluids can transport acoustic waves in longitudinal mode, only. However, for the fiber application, the transverse mode is applicable. So, we can conclude that fluids are not applicable for phononic crystal fiber designing and only solid–solid phononic crystals are useful for realization of phononic crystal fibers. The classifications of photonic crystal fibers are index-guiding and photonic band gap-guiding fibers^1^. Our design is based on phononic band gap-guiding class where this fiber class is realizable.

In the following, design procedure of phononic crystal fiber will be presented in “Design procedure of phononic crystal fiber” section. Simulation results are come and discussed in “Simulation results and discussions” section. Finally, results and discussions will be concluded in “Conclusions” section.

## Design procedure of phononic crystal fiber

**Figure 1:**
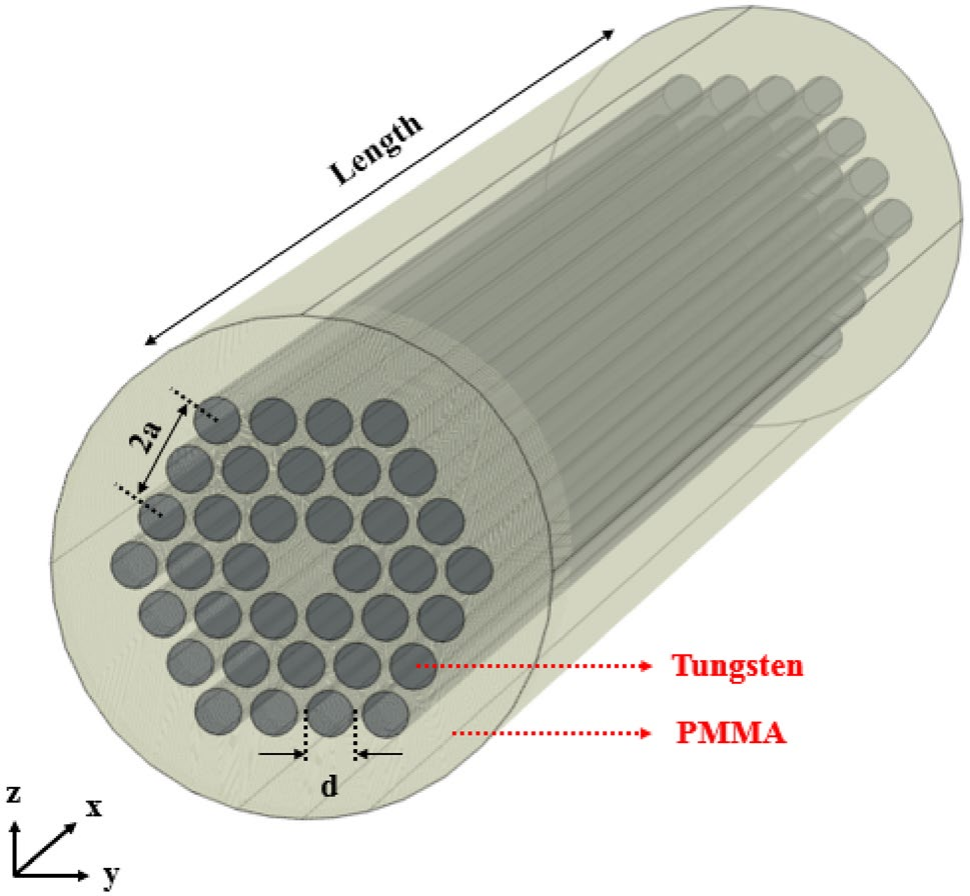
Schematics of proposed phononic crystal fiber and its constituent materials.

The first step in the most phononic crystal designs is to achieve the phononic band gap. By utilizing two different materials with high elastic contrast, phononic band gap or prohibited region appears in the transmission spectrum of the periodic structure. In this design, tungsten rods have been embedded in the PMMA background with hexagonal arrangement. The periodic structure operates as cladding and core region has been formed by eliminating one or more central rods. Total schematics of proposed phononic fiber has been shown in Fig. 1.

According to Fig. 1, tungsten rods are embedded in PMMA background with hexagonal arrangement. Table 1 illustrates elastic properties of rod and background materials.

**Table 1:** The elastic properties of rod and background materials of proposed phononic crystal fiber.

Material	Mass density (kg/m^3^)	Young’s modulus (GPa)	Poisson ratio
PMMA	1190	3	0.35
Tungsten	19,350	411	0.28

**Figure 2:**
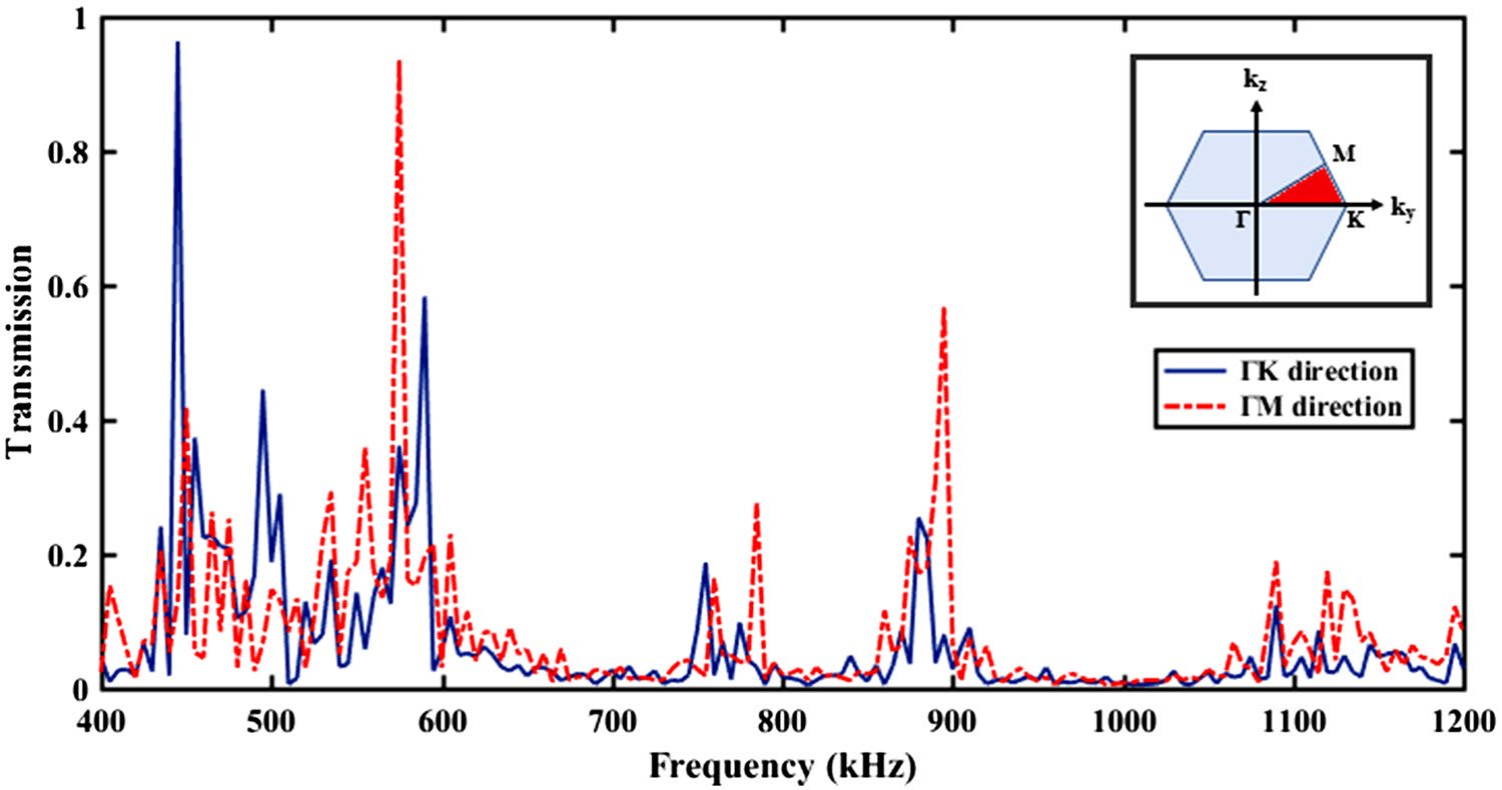
Transmission spectrum of phononic crystal fiber without core when input wave has only y-component for achieving the transmission in ΓK direction, and only z-component for achieving the transmission in ΓM direction of hexagonal Brillouin zone.

Diameter of rods is named as *d* and equal to 1 mm. The distance between rod centers has been considered equal to a = 1.2 mm. In order to confine the applied elastic wave in the core region, the periodic structure in the surrounding of core is considered. Transmission spectrum of designed phononic crystal fiber with any core has been calculated based on finite element method (FEM) in the length of 4 mm (which is more than wavelength corresponding to all spectrum frequencies) and its result has been depicted in Fig. 2.

In order to obtain the transmission spectrum in two different directions of hexagonal lattice, once incident wave has been applied with y-component, only. Then, the incident wave has been applied with z-component. Input source is displacement excitation with plane wave type propagation considered as one of the boundary conditions on the input cross-section of phononic crystal fiber. Also, another boundary condition has been considered as free. To avoid of divergent responses, two points in the beginning and the end of the fiber structure have been considered as fixed. From Fig. 1 and inset of Fig. 2, it is clear that input wave with y-component can oscillate in ΓK direction and with z-component can oscillate in ΓM direction of hexagonal lattice Brillouin zone. Since phononic crystal periodicity exists in the cross section of phononic fiber, so transverse waves can sense it, only. On the other words, prohibited frequency region in the transmission curve are related to transverse waves means longitudinal elastic waves cannot sense phononic crystal periodicity. Thus, in the phononic crystal fibers only transverse waves are applicable. According to the Fig. 2, band gap frequency regions for y-component have been located in 400 kHz to 430 kHz, 610 kHz to 750 kHz, 780 kHz to 865 kHz and 915 kHz to 1085 kHz. Also, band gap frequency regions for z-component have been located in 410 kHz to 430 kHz, 610 kHz to 755 kHz, 790 kHz to 870 kHz and 915 kHz to 1085 kHz.

The fundamental equations to describe the elastic wave propagation are defined as follows^3^:

1 $$ T_{ij} (\vec{r},t) = c_{ijkl} (\vec{r})u_{k,l} (\vec{r},t) $$

2 $$ T_{ij,j} (\vec{r},t) = \rho (\vec{r})\frac{{\partial^{2} u_{i} (\vec{r},t)}}{{\partial t^{2} }} $$

where *T*_*ij*_ and *u*_*i*_ are stress tensor and three displacements in space directions, respectively. Also, mass density and elastic constants are defined as $\rho$ and *c*_*ijkl*_, respectively. Utilized indices such as *i*, *j*, *k*, and *l* run from 1 to 3 for space directions. A comma before an index illustrates the derivation (e.g., $u_{k,l} = \frac{{\partial u_{k} }}{{\partial x_{l} }}$), and summation over repeated indices is implied (e.g., $T_{ij,j} = \sum\nolimits_{j = 1}^{3} {\frac{{\partial T_{ij} }}{{\partial x_{j} }}}$). By combination of Eqs. 1 and 2, the following equation is obtained^3^:

3 $$ (c_{ijkl} (\vec{r})u_{k,l} (\vec{r},t))_{,j} = \rho (\vec{r})\frac{{\partial^{2} u_{i} (\vec{r},t)}}{{\partial t^{2} }} $$

The last equation is used as the fundamental equation for modeling the solid–solid phononic crystals without stresses.

## Simulation results and discussions

**Figure 3:**
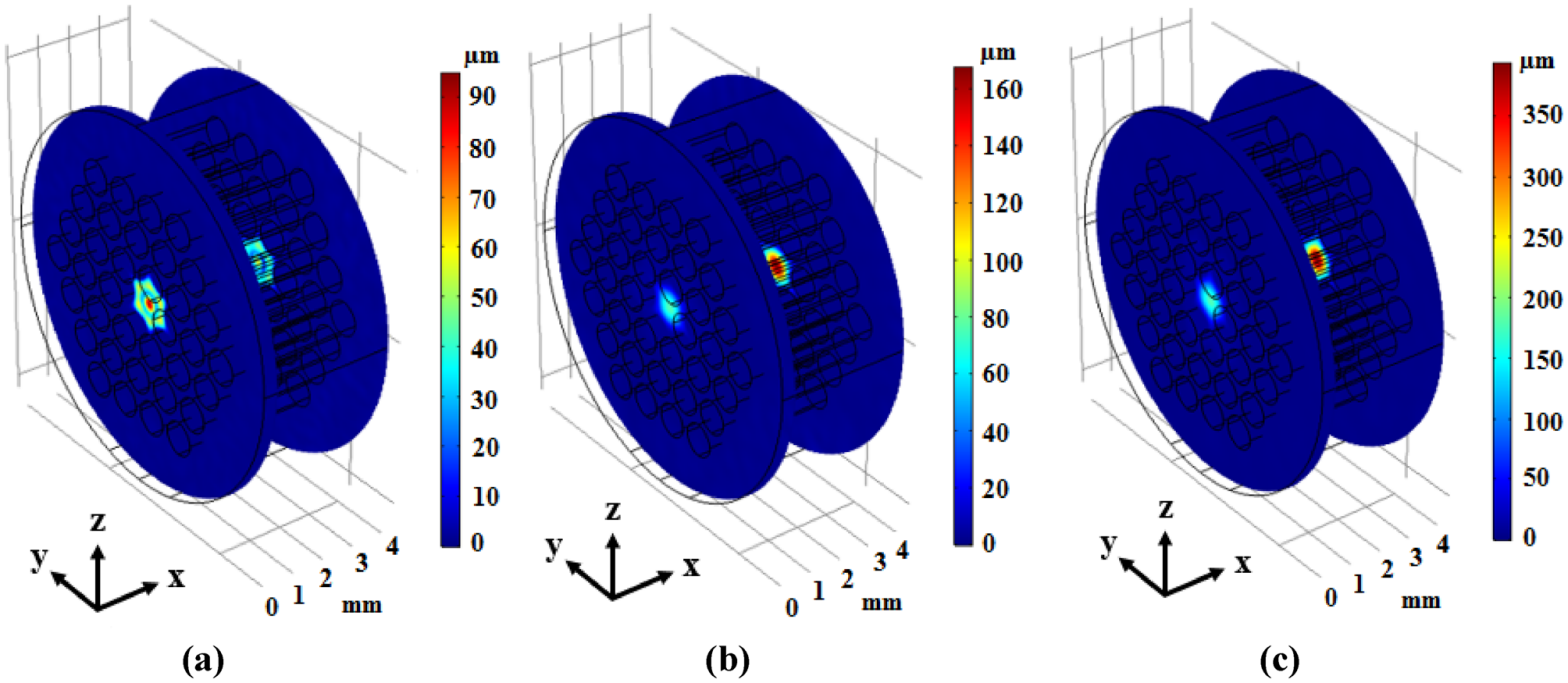
Displacement field of phononic crystal fiber with small core corresponding to incident waves with (**a**) x-component and frequency of 1158.1 kHz, (**b**) y-component and frequency of 738.9 kHz, and (**c**) z-component and frequency of 738.9 kHz in two input and output cut-planes.

A novel phononic crystal fiber with design procedure discussed in the previous section has been proposed. In this section, simulation results are presented. Elastic transverse wave oscillation in the core region of fiber is similar to cavity in the cross-section view. So, it is important to achieve the resonance frequency of this cavity. Elastic wave propagation has been calculated for phononic crystal fiber shown in Fig. 1, and its results can be seen in Fig. 3.

**Figure 4:**
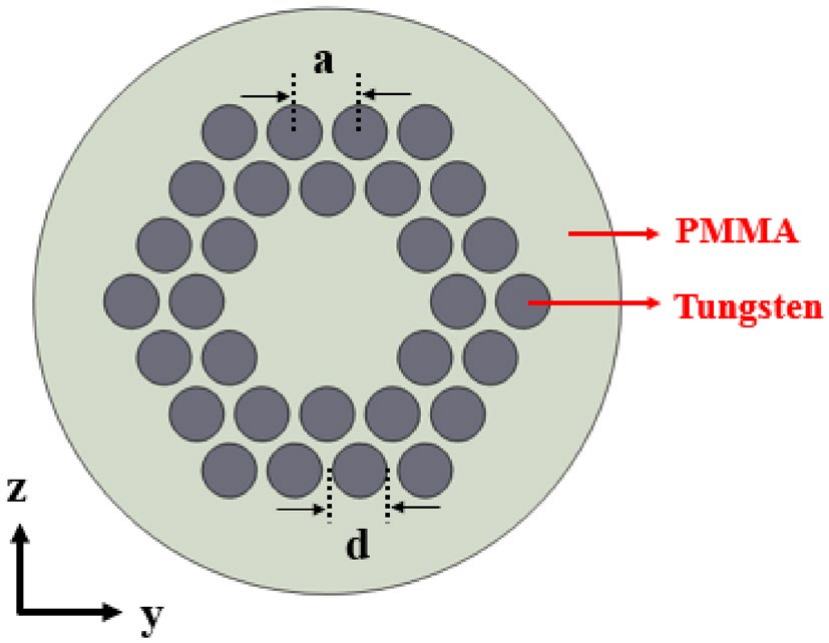
The circular cross section of phononic crystal fiber with large core.

According to the Fig. 3, confinement of incident elastic wave with x-component and 1158.1 kHz frequency can be seen in (a) part. Also, input waves with y-component and frequency of 738.9 kHz can be confined in the core region of fiber with very high confinement which has been shown in (b) part of Fig. 3. Then, the incident elastic waves with z-component and frequency of 738.9 kHz have been confined in center of fiber and propagated in the fiber length direction as shown in Fig. 3c. The confinement frequency is related to the core dimension. Also, a phononic crystal fiber with large core formed by removing a single ring of rods around the central tungsten rod. The circular cross section of this large core phononic crystal fiber has been depicted in Fig. 4.

**Figure 5:**
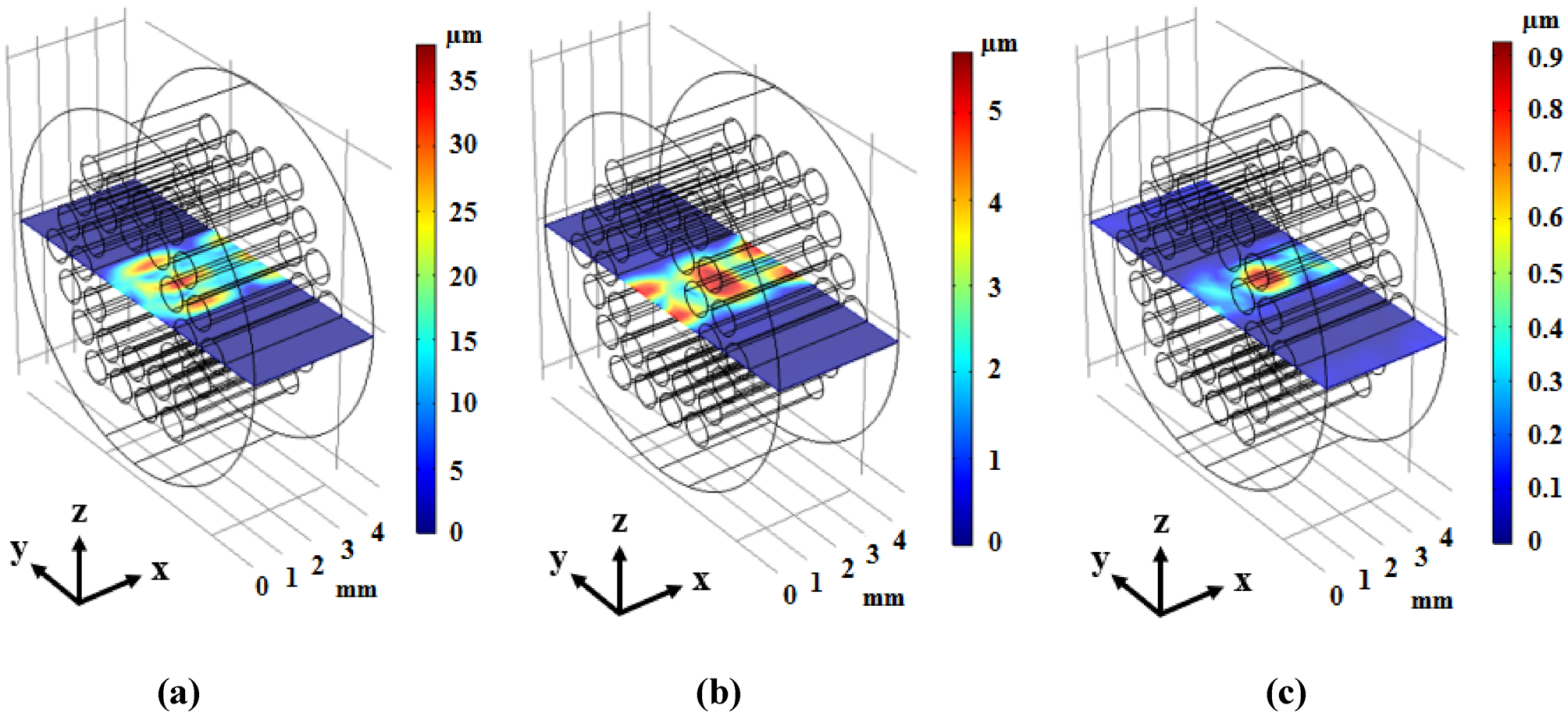
Displacement field of the phononic crystal fiber with large core corresponding to incident waves with (**a**) x-component and frequency of 524 kHz, (**b**) y-component and frequency of 424.5 kHz, and (**c**) z-component and frequency of 426 kHz in the cut-plane.

The propagation of incident elastic waves in the phononic crystal fiber with large core has been calculated and its results have been shown in Fig. 5.

According to Fig. 5, the confinement of incident elastic wave with x-component and 524 kHz frequency can be seen in Fig. 5a. Also, input waves with y-component and frequency of 424.5 kHz can be confined in core region of fiber which has been shown in (b) part of Fig. 5. Finally, the incident elastic waves with z-component and frequency of 426 kHz have been confined in the center of fiber and propagated in the fiber length direction as shown in Fig. 5c. It can be seen that solid–solid phononic crystals as cladding confine and transmit incident elastic waves with very high confinement in the core region. Hence, we can conclude that phononic crystal fibers can be realized for acoustic field and related applications. This discovery of phononic crystal fibers is a very novel and applicable idea in the sonic communications. One of the important properties of fibers is the high confinement capability in their core region. So, it is useful to define a suitable parameter which can indicate the confinement capability of the fibers. In this way, we have defined a confinement parameter as follows:

4 $$ Ideality\;Confinement\;Factor = \frac{{U_{core} }}{{U_{cladding} }} $$

**Figure 6:**
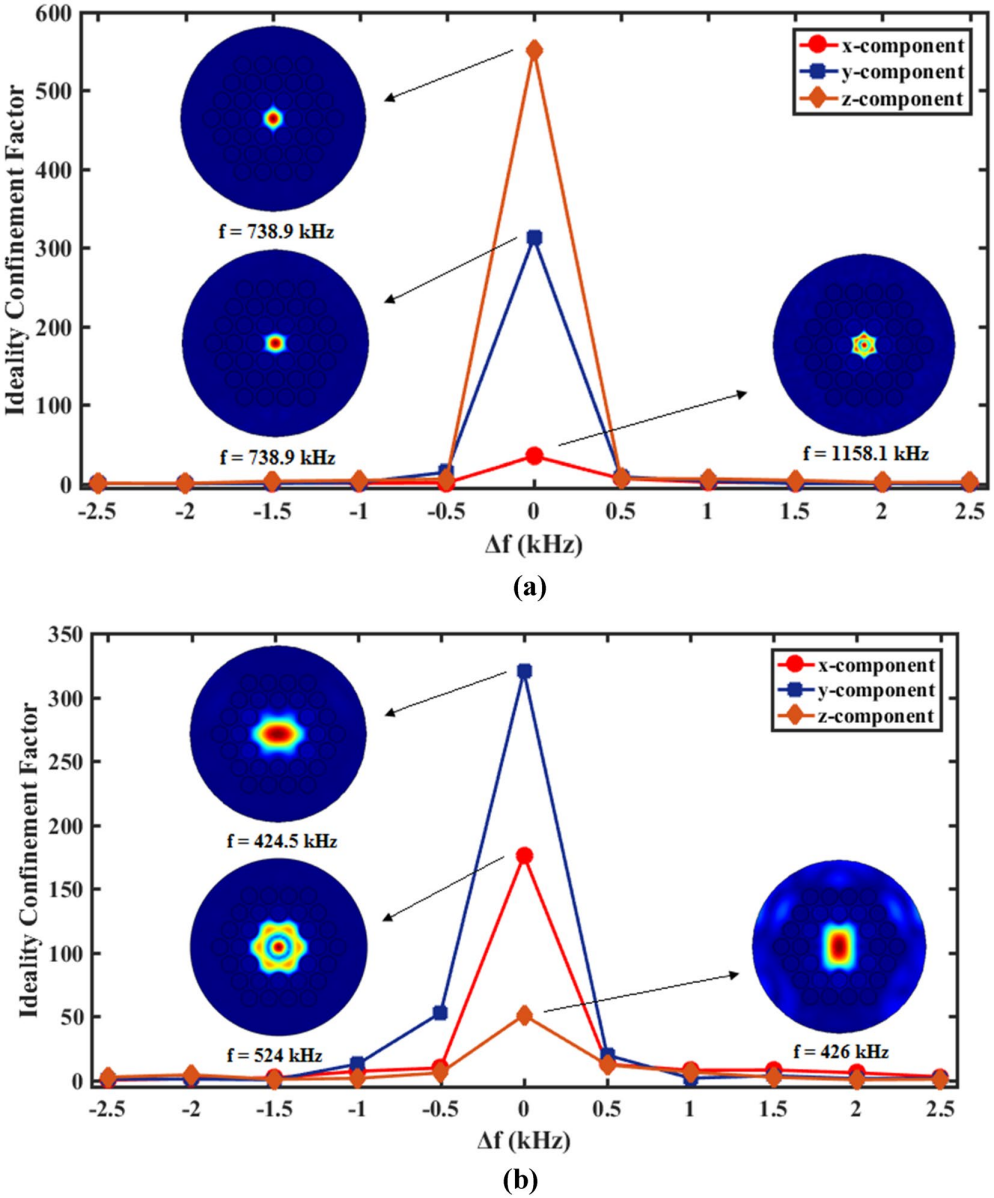
The confinement quality factor of designed phononic crystal fibers with (**a**) small and (**b**) large in the 2.5 kHz frequency range around the resonance frequencies.

Regarding the Eq. (4), the maximum displacement in the core region of fiber is $U_{core}$ and the maximum displacement in the surroundings of core is $U_{cladding}$. According to the defined factor, it is clear that increasing the wave displacement (wave confinement) in the core increase the ideality confinement factor. This factor can be decreased due to increase the elastic wave displacement in the cladding limits. Hence, high ideality confinement factor indicates high quality performance of phononic crystal fibers. The confinement quality of designed phononic crystal fibers with small and large core has been investigated in the 2.5 kHz frequency range around the resonance frequencies which mentioned in Figs. 3 and 5. Results of this investigation has been shown in Fig. 6.

According to Fig. 6a, the ideality confinement factor has been calculated for phononic crystal fiber with small core when incident waves applied with x, y, and z-components, separately. In this curve, the maximum confinement factors are related to the previously designed frequencies. The variations of $\Delta f$ have been considered around the depicted frequencies on the Fig. (1158.1, 738.9 and 738.9 kHz corresponded to input waves with x-, y- and z-components, respectively). The inset figures of displacement field are related to $\Delta f = 0\;{\text{kHz}}$ and x, y and z-component of applied elastic waves. Also, the ideality confinement factor has been calculated for phononic crystal fiber with large core in Fig. 6b. These results indicate that proposed phononic crystal fibers confine the applied elastic waves. Another important property of fibers is their longitudinal losses. By assuming damping ratio for PMMA core, $\eta = 0.02$ ^15^, the maximum longitudinal loss in proposed phononic crystal fibers is equal to 0.35 dB/km. So, phononic crystal fibers similar to photonic crystal fibers can be realized.

It should be mentioned that the proposed phononic crystal fiber can used in sonar applications which are related to the sonic communication field. Also, can operate as sensor and detector devices for sensing and detecting applications.

## Conclusions

In this work, we have tried to design a novel phononic crystal fiber based on solid–solid phononic crystal as cladding for the first time. In the proposed fiber structure, solid–solid phononic crystal has been used, only; because in fluids, the longitudinal polarization can only propagate. However, on the other side, the periodicity of phononic crystal is radial and transverse waves can sense this periodicity. So, solid–solid phononic crystals are an appropriate choice for our purpose. Simulation results of proposed phononic fiber can indicate that applied elastic waves into the fiber can be confined and propagated in the core region with low losses. So, we conclude that phononic crystal fiber is realizable.

## Data Availability

The data that support the findings of this study are available from the corresponding author upon reasonable request.
